# Combined Usefulness of the Platelet-to-Lymphocyte Ratio and the Neutrophil-to-Lymphocyte Ratio in Predicting the Long-Term Adverse Events in Patients Who Have Undergone Percutaneous Coronary Intervention with a Drug-Eluting Stent

**DOI:** 10.1371/journal.pone.0133934

**Published:** 2015-07-24

**Authors:** Kyoung Im Cho, Soe Hee Ann, Gillian Balbir Singh, Ae-Young Her, Eun-Seok Shin

**Affiliations:** 1 Department of Cardiology, Kosin University School of Medicine, Busan, South Korea; 2 Department of Cardiology, Ulsan University Hospital, University of Ulsan College of Medicine, Ulsan, South Korea; 3 Division of Cardiology, Department of Internal Medicine, School of Medicine, Kangwon National University, Chuncheon, South Korea; Virginia Commonwealth University, UNITED STATES

## Abstract

**Objectives:**

The aim of this study was to investigate the combined usefulness of platelet-to-lymphocyte ratio (PLR) and neutrophil-to-lymphocyte ratio (NLR) in predicting the long-term adverse events in patients who have undergone percutaneous coronary intervention (PCI) with a drug-eluting stent (DES).

**Methods:**

798 patients with stable angina, unstable angina and non-ST elevated myocardial infarction (NSTEMI) who underwent elective successful PCI with DES were consecutively enrolled. The value of PLR and NLR in predicting adverse coronary artery disease (CAD) events and the correlations between these markers and adverse events (all-cause mortality, cardiac death, and nonfatal myocardial infarction) were analyzed.

**Results:**

The follow-up period was 62.8 ± 28.8 months. When patients were classified into four groups according to the optimal cut-off values for the PLR and NLR on receiver operating characteristic analysis, patients with a high PLR (>128) and high NLR (>2.6) had the highest occurrence of adverse events among the groups. On Cox multivariate analysis, the NLR >2.6 [hazard ratio (HR) 2.352, 95% confidence interval (CI) 1.286 to 4.339, p = 0.006] and the PLR >128 (HR 2.372, 95% CI 1.305 to 3.191, p = 0.005) were independent predictors of long-term adverse events after adjusting for cardiovascular risk factors. Moreover, both a PLR >128 and a NLR >2.6 were the strongest predictors of adverse events (HR 2.686, 95% CI 1.452 to 4.970, p = 0.002).

**Conclusion:**

High pre-intervention PLR and NLR, especially when combined, are independent predictors of long-term adverse clinical outcomes such as all-cause mortality, cardiac death, and myocardial infarction in patients with unstable angina and NSTEMI who have undergone successful PCI with DES.

## Introduction

Previous studies have shown that inflammatory response plays an important role in the progression and destabilization of atherosclerosis and cardiovascular diseases [1,2]. Among the various inflammatory markers, the white blood cell count and its subtypes are associated with increased cardiovascular risk factors [3,4]. Recently, the neutrophil-to-lymphocyte ratio (NLR), which is inexpensive, routinely used, reproducible, and widely available in most hospitals, has been proven to be an important inflammatory marker and potential predictor of cardiovascular risk [5,6]. Although patients with ST-segment elevation myocardial infarction (MI) show a strong association between NLR and cardiovascular events including all-cause mortality, few studies have shown an association between NLR and adverse clinical outcomes in patients undergoing elective cardiac revascularization [7–9]. As increased platelet activation plays a major role in the initiation and progression of atherosclerosis [10], recent studies have also shown the platelet-to-lymphocyte ratio (PLR) to be a new inflammatory marker and predictor of adverse outcomes in various cardiovascular diseases [11–13]. Moreover, a high pre-procedural PLR is reported to be a significant independent predictor of long-term mortality in acute coronary syndrome (ACS) [14,15]. The combined usefulness of PLR and NLR in predicting the long-term adverse events in coronary artery disease (CAD), however, has not been sufficiently evaluated. The aim of the present study was to investigate the combined usefulness of PLR and NLR in predicting the long-term clinical outcomes in patients who have undergone percutaneous coronary intervention (PCI) with a drug-eluting stent (DES).

## Materials and Methods

### Study population

All consecutive eligible patients hospitalized at our institution between March 2003 and August 2007 due to stable angina pectoris or ACS (unstable angina and non-ST elevated myocardial infarction, NSTEMI) who underwent successful PCI with DES were retrospectively enrolled in this study. NSTEMI was defined as an increased value for cardiac troponin-T or CK-MB defined as a measurement exceeding the 99th percentile of a normal reference population on first assessment and at 6–9 hours later together with symptoms of ischemia, without typical ST elevation in electrocardiography [16].

Patients with systemic diseases and on treatments potentially affecting the white blood cell count, including hematological disorders, malignancies, chemotherapy treatment, evidence of concomitant inflammatory disease, acute infection, chronic inflammatory conditions, history of corticosteroid therapy in the preceding 3 months, history of previous PCI or coronary artery bypass graft, secondary hypertension, heart failure, history of chronic renal or hepatic disease, and cerebrovascular disease were excluded from the study. We defined chronic renal disease as eGFR< 30 ml/min/1.73m^2^. Out of the 994 eligible patients, 187 patients undergoing primary PCI for ST-segment elevation MI (STEMI) and 6 patients with unavailable laboratory data were excluded. Three patients were lost to follow-up after discharge from the hospital. Therefore, the remaining cohort consisted of 798 patients. This study protocol was approved by the Ulsan University Hospital Institutional Review Board (IRB) ethics committee and written informed consent was obtained from all participants.

### Study procedures

Complete blood counts, which included the total white blood cells, neutrophils, lymphocytes, and platelets, were obtained from venous sampling at the time of admission. Cardiac enzymes (CK-MB and high sensitivity troponin T), glucose, creatinine, lipid profiles, and high sensitivity C-reactive protein (hs-CRP) were also measured in all patients. The PLR was calculated as the ratio of the platelet count to the lymphocyte count and the NLR was calculated as the ratio of the neutrophil count to the lymphocyte count. All patients were evaluated for the presence of cardiovascular risk factors.

All patients received a 200 mg loading dose of aspirin, a 300 mg or 600 mg loading dose of clopidogrel at least 12 hours before the DES implantation, and an intravenous dose of unfractionated heparin (8000 IU or 100 IU/kg) after arterial puncture. Stents were implanted according to standard techniques. A successful PCI was defined as the attainment of an angiographic residual stenosis diameter of less than 30% and an antegrade flow of TIMI 3. Post PCI patients without contraindications remained on aspirin indefinitely and clopidogrel for at least 12 months.

### Study definitions and endpoints

The primary endpoints were all-cause mortality, cardiac death, and nonfatal MI. Secondary outcomes were stroke, target vessel revascularization (TVR) and target lesion revascularization (TLR) based on the Academic Research Consortium (ARC) definition [17]. Cardiac death was defined as death resulting from an evident cardiac cause or any death related to PCI. Nonfatal MI was defined according to the European Society of Cardiology, American College of Cardiology (ACC), American Heart Association (AHA), and World Heart Federation definitions [16]. TVR was defined as any clinically-driven repeat PCI or surgical bypass of any segment within the entire epicardial coronary artery containing the target lesion. TLR was defined as any clinically driven repeat revascularization caused by a 50% stenosis within the stent or within a 5-mm border proximal or distal to the stent.

### Statistical analysis

The Kolmogorov-Smirnov test was used for the evaluation of variable distribution. Continuous data with normal distributions were expressed as mean ± standard deviation, while categorical data is presented as the number of patients (%). The Chi-square test was used for comparison of categorical variables. We compared demographic characteristics and variables among the four groups using ANOVA tests for continuous variables and the Tukey method for post-hoc analysis. Receiver operating characteristic (ROC) curves were used to differentiate the ability of the PLR and NLR to predict adverse events (all-cause mortality, cardiac death, nonfatal MI). Cutoff values, sensitivity, and specificity were derived for each parameter. Survival curves were constructed based on cumulative incidences with Kaplan–Meier estimates and compared using the log rank test. The efficacy of the PLR and NLR in predicting adverse events was analyzed by univariate and multivariate Cox proportional hazards regression analyses adjusted for the variables with a significance level of p < 0.10 in univariate analysis. The statistical significance threshold was set at p < 0.05. Statistical analyses were performed using SPSS version 18.0 (SPSS Inc., Chicago, IL, USA).

## Results

A total of 798 patients (64% male, mean age 60.7 ± 10.1 years, age range 29–86 years) were included in the study and the median follow-up period was 62.8 ± 28.8 months. In total 51 adverse events occurred during the follow-up period, 5 adverse events occurred in 90 patients with stable angina and 46 adverse events occurred in 708 ACS patients (562 patients with unstable angina and 146 patients with NSTEMI). Among the 798 patients, 706 patients completed 1 year follow-up (92 patients were under 1 year and there were 16 deaths). Thirty-six patients had between 1–2 years follow-up, and a total of 670 patients completed both the 1 year and 2 years follow-up. Patients lost to follow-up (n = 112) were excluded from the analysis.

On ROC analysis, the PLR and NLR were found to have the largest area under the curve (AUC = 0.605, 95% confidence interval [CI] 0.570 to 0.639, p = 0.018 and AUC = 0.633, 95% CI 0.599 to 0.667, p = 0.003, respectively) with an optimal PLR cut-off value of 128 (sensitivity 56%, specificity 65%) and an optimal NLR cut-off value of 2.6 (sensitivity 52%, specificity 75%) for predicting adverse events.

Patients were classified into four groups based on the optimal cut-off values of the PLR and NLR: a low PLR and a low NLR (PLR <128, NLR <2.6, n = 438), a high PLR and a low NLR (PLR >128, NLR <2.6, n = 147), a low PLR and a high NLR (PLR <128, NLR >2.6, n = 70), and a high PLR and a high NLR (PLR >128, NLR >2.6, n = 143). Table 1 shows the baseline clinical characteristics of the study population according to the four groups while Table 2 shows the laboratory findings for each group. Baseline characteristics and clinical data were similar among groups except that patients with a high PLR and a high NLR who were significantly older, were less likely to be a current smoker, had a lower BMI, higher hs-CRP, higher creatinine, lower eGFR, and lower triglycerides (Tables 1 and 2). Although patients with a high PLR and a high NLR have significantly reduced LV ejection fraction compared to the other groups (Table 1), LV ejection fraction was within normal values in all groups.

**Table 1:** Baseline clinical characteristics of the study population according to neutrophil-to-lymphocyte ratio (NLR) and platelet-to-lymphocyte ratio (PLR).

	Low PLR, Low NLR (n = 438)	High PLR, Low NLR (n = 147)	Low PLR, High NLR (n = 70)	High PLR, High NLR (n = 143)	p-value
Age, years	60.1 ± 10.0	60.8 ± 9.70	61.1 ± 10.5	62.8 ± 10.4*	0.049
Age>65, n (%)	161 (36.8)	55 (37.4)	25 (35.7)	73 (51) *	0.018
BMI, kg/m^2^	24.7 ± 2.94	24.4 ± 3.01	24.3 ± 2.84	23.6 ± 3.35*	0.008
Systolic BP, mmHg	129.2 ± 19.9	126.5 ± 20.1	125.0 ± 20.1	126.0 ± 18.5	0.145
Diastolic BP, mmHg	78.5 ± 11.1	76.4 ± 11.8	77.0 ± 12.8	76.2 ± 11.4	0.073
Male, n (%)	284 (64.8)	82 (55.8)^+^	53 (75.7)	95 (66.4)	0.030
Current smoker, n (%)	191 (43.6)	40 (27.2)	33 (47.1)	41 (28.7)	<0.001
Hypertension, n (%)	228 (52.1)	70 (47.6)	27 (38.6)	76 (53.1)	0.153
Diabetes mellitus, n (%)	112 (25.6)	27 (18.4)	18 (25.7)	39 (27.3)	0.272
Dyslipidemia, n (%)	216 (49.3)	61 (41.5)	35 (50)	56 (39.2)	0.099
Family history of CAD, n (%)	5 (1.1)	1 (1.4)	0	2 (1.4)	0.758
Previous MI, n (%)	18 (4.1)	5 (3.4)	1 (1.4)	7 (4.9)	0.643
Ejection fraction, %	61.3 ± 8.03	62.2 ± 7.37	57.3 ± 9.46*	58.7 ± 10.2* ^,^ ^#^	<0.001
Discharge medication					
Beta blocker, n (%)	221 (50.5)	78 (53.1)	34 (48.6)	71 (49.7)	0.914
CCB, n (%)	96 (21.9)	32 (21.8)	12 (17.1)	37 (25.9)	0.535
Nitrate, n (%)	308 (70.3)	99 (67.3)	43 (61.4)	89 (62.2	0.191
ACE inhibitor, n (%)	199 (45.4)	70 (47.6)	26 (37.1)	68 (47.6)	0.323
ARB, n (%)	28 (6.4)	8 (5.4)	3 (4.3)	8 (5.6)	0.894
Statin, n (%)	281 (64.2)	96 (65.3)	46 (65.7)	83 (58)	0.510
Aspirin, n (%)	432 (98.6)	142 (96.6)	69 (98.6)	141 (98.6)	0.310
Clopidogrel, n (%)	422 (96.3)	145 (98.6)	65 (92.9)	139 (97.2)	0.160

Data is presented as mean ± SD or number (percentage)

BMI body mass index, BP blood pressure, CAD coronary artery disease, CCB calcium channel blocker, ACE angiotensin converting enzyme, ARB angiotensin receptor blocker

*: p<0.05 compared with Low PLR, Low NLR)

+: p<0.05 compared with Low PLR, High NLR

#: p<0.05 compared with High PLR, Low NLR

**Table 2:** Baseline laboratory characteristics of the study population according to neutrophil-to-lymphocyte ratio (NLR) and platelet-to-lymphocyte ratio (PLR).

	Low PLR, Low NLR (n = 438)	High PLR, Low NLR (n = 147)	Low PLR, High NLR (n = 70)	High PLR, High NLR (n = 143)	p-value
White blood cell, x10^9^/L	7.56 ± 1.93	6.11 ± 1.51*	10.31±3.04*	8.65 ± 2.96* ^,^ ^+^ ^,^ ^#^	<0.001
Neutrophil, %	52.4 ± 7.79	57.4 ± 5.35*	76.7 ± 58.4*	72.6 ± 7.20* ^,^ ^#^	<0.001
Lymphocyte,%	36.1 ± 7.04	30.9 ± 4.55*	21.4 ± 3.55*	17.9 ± 5.02* ^,^ ^+^ ^,^ ^#^	<0.001
Monocyte, %	5.30 ± 1.57	5.52 ± 1.90	4.85 ±0.45	4.84 ± 1.90* ^,^ ^#^	0.001
Eosinophil, %	3.57 ± 2.92	3.52 ± 2.50	2.30 ± 2.21*	2.73 ± 2.71*	<0.001
Hemoglobin, g/dL	13.8 ± 1.56	13.0 ± 1.61*	14.4 ± 3.54	12.9 ± 1.86* ^,^ ^+^	<0.001
Hematocrit, %	39.7 ± 4.47	37.8 ± 4.41*	40.1 ± 4.29	37.4 ± 5.12* ^,^ ^+^	<0.001
Platelets, x10^9^/L	241.3 ± 56.7	303.9 ± 78.2*	213.7 ± 58.3*	274.8 ± 78.1* ^,^ ^+^ ^,^ ^#^	<0.001
Platelet density width, %	51.7 ± 3.44	52.6 ± 2.87*	50.8 ± 3.45	52.4 ± 2.88^+^	<0.001
NLR	1.54 ± 0.49	1.91 ± 0.40*	3.63 ± 1.87*	4.74 ± 2.79* ^,^ ^+^ ^,^ ^#^	<0.001
PLR	93.0 ± 20.9	166.4 ± 39.3*	101.0 ± 22.1	197.1 ± 63.9* ^,^ ^+^ ^,^ ^#^	<0.001
hs-CRP, mg/dl	0.46 ± 2.14	0.61 ± 1.86	1.36 ± 2.84	1.60 ± 3.41* ^,^ ^#^	<0.001
Hemoglobin A1c, %	6.78 ± 1.49	6.46 ± 1.56	6.59 ± 1.37	6.44 ± 1.35	0.364
Creatinine, mg/dl	1.10 ± 0.33	1.08 ± 0.23	1.15 ± 0.39	1.21 ± 0.43* ^,^ ^#^	0.001
eGFR, ml/min/1.73m^2^	69.5 ± 16.4	70.1 ± 15.2	72.0 ± 20.9	65.6 ± 17.1* ^,^ ^#^	0.026
Total Cholesterol, mg/dl	194.4 ± 42.2	190.1 ± 47.5	191.4 ± 39.3	186.6 ± 41.4	0.261
LDL, mg/dl	116.7 ± 38.3	115.1 ± 37.0	111.2 ± 36.6	114.7 ± 36.8	0.736
HDL, mg/dl	41.9 ± 16.3	46.2 ± 28.1	45.9 ± 30.0	46.8 ± 56.1	0.255
Triglycerides, mg/dl	157.5 ± 111.8	142.0 ± 89.3	159.0 ± 107.2	120.5 ± 67.7*	0.002

Data is presented as mean ± SD or number (percentage)

NLR neutrophil to lymphocyte ratio, PLR platelet to lymphocyte ratio, hs-CRP high sensitivity C-reactive protein, eGFR estimated glomerular filtration rate according to the Modification of Diet in Renal Disease (MDRD) equation, LDL low density lipoprotein, HDL high density lipoprotein.

*: p<0.05 compared with Low PLR, Low NLR

+: p<0.05 compared with Low PLR, High NLR

#: p<0.05 compared with High PLR, Low NLR

Medications prior to PCI, both during the in-hospital period and at discharge, including antiplatelets (aspirin and/or clopidogrel), beta-blockers, renin-angiotensin-aldosterone system blockers, and statins, were similar among the groups. The angiographic characteristics based on groups are shown in Table 3, and there were no significant differences in the presence of ACC/AHA B2C lesions, number of stents, type of stents, stent length, and stent diameter among the groups.

**Table 3:** Angiographic characteristics of the study population according to neutrophil-to-lymphocyte ratio (NLR) and platelet-to-lymphocyte ratio (PLR)

	Low PLR, Low NLR (n = 438)	High PLR, Low NLR (n = 147)	Low PLR, High NLR (n = 70)	High PLR, High NLR (n = 143)	p-value
Indication for PCI, n (%)		<0.001			
Stable angina	52 (11.9)	17 (11.6)	3 (4.3)	18 (12.6)	
Unstable angina	319 (72.8)	116 (78.9)	38 (54.3)	89 (62.2)	
NSTEMI	67 (15.3)	14 (9.5)	29 (41.4)	36 (25.2)	
CAD		0.399			
1-vessel, n (%)	192 (43.8)	70 (47.6)	35 (50)	56 (39.2)	
2-vessel, n (%)	174 (39.7)	56 (38.1)	21 (30)	56 (39.2)	
3-vessel /Left main, n (%)	71 (16.2)	21 (14.3)	14 (20)	31 (21.7)	
Number of target lesions		0.622			
1, n (%)	303 (69.2)	106 (72.1)	51 (72.9)	99 (69.2)	
2, n (%)	111 (25.3)	30 (20.4)	17 (24.3)	31 (21.7)	
3, n (%)	21 (4.8)	11 (7.5)	2 (2.9)	12 (8.4)	
>3, n (%)	2 (0.5)	0	0	1 (0.7)	
ACC/AHA B2/C lesion, n (%)	279 (63.7)	89 (60.5)	49 (70)	97 (67.8)	0.474
Number of DES	1.51 ± 0.79	1.57 ± 0.79	1.67 ± 0.93	1.58 ± 0.87	0.402
Type of DES, n (%)					
Sirolimus eluting stent	387 (88.3)	121 (82.3)	63 (90)	126 (88.1)	0.231
Paclitaxel eluting stent	44 (10)	20 (13.6)	4 (5.7)	14 (9.7)	0.435
Total stent length, mm	39.4 ± 22.9	41.4 ± 24.7	45.2 ± 27.3	40.8 ± 24.2	0.218
Stent diameter, mm	3.15 ± 0.31	3.19 ± 0.32	3.17 ± 0.33	3.29 ± 1.68	0.345

Data is presented as mean ± SD or number (percentage)

NLR neutrophil to lymphocyte ratio, PLR platelet to lymphocyte ratio, PCI percutaneous coronary intervention, CAD coronary artery disease, NSTEMI non ST-segment elevation myocardial infarction, DES drug eluting stent

**Fig 1:**
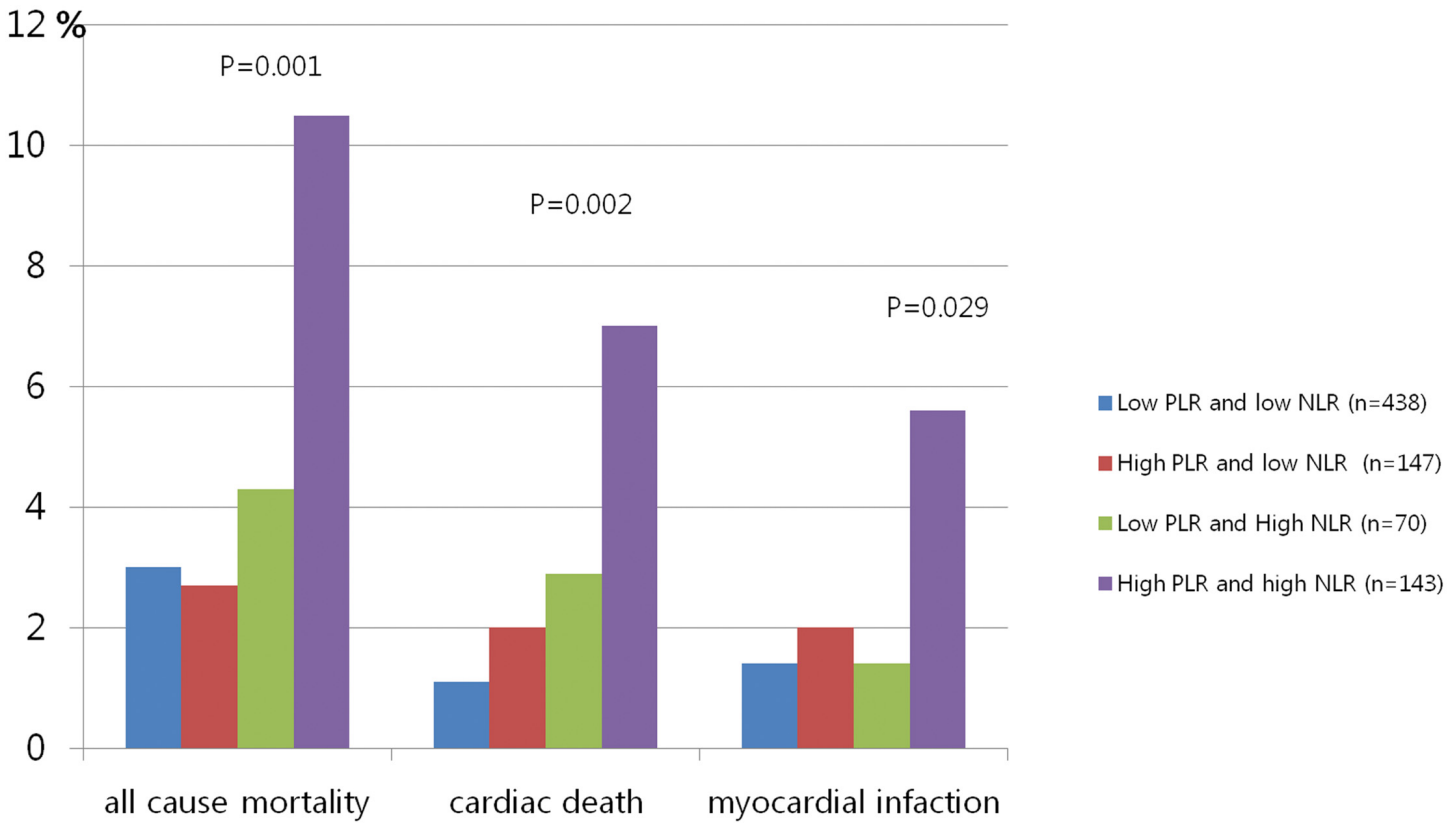
Comparison of all-cause mortality, cardiac death, and nonfatal MI among the groups.

All-cause mortality, cardiac death, and nonfatal MI, as well as the composite primary endpoints, were significantly higher in patients with a high PLR and high NLR compared to the patients with a low PLR and low NLR (Table 4, Fig 1). There were no significant differences in TLR and TVR among the groups. Table 5 shows that both the NLR and PLR were significantly associated with age, body mass index, hs-CRP, stent diameter, all-cause mortality, and cardiac death. LV ejection fraction and nonfatal MI were only significantly associated with NLR.

**Table 4:** Clinical outcomes of the study population according to neutrophil-to-lymphocyte ratio (NLR) and platelet-to-lymphocyte ratio (PLR).

	Low PLR, Low NLR (n = 438)	High PLR, Low NLR (n = 147)	Low PLR, High NLR (n = 70)	High PLR, High NLR (n = 143)	p-value
Primary end point, n (%)	19 (4.3)	7 (4.7)	4 (5.7)	23 (16.1) * ^,^ ^+^ ^,^ ^#^	<0.001
All-cause mortality, n (%)	13 (3.0)	4 (2.7)	3 (4.3)	15 (10.5) * ^,^ ^#^	0.001
Cardiac death, n (%)	5 (1.1)	3 (2.0)	2 (2.9)	10 (7.0) * ^,^ ^#^	0.002
Nonfatal MI, n (%)	6 (1.4)	3 (2.0)	1 (1.4)	8 (5.6) * ^,^ ^#^	0.029
Stroke, n (%)	6 (1.4)	2 (1.4)	2 (2.9)	3 (2.1)	0.777
TLR, n (%)	45 (10.3)	15 (10.2)	8 (11.4)	19 (13.3)	0.768
TVR, n (%)	10 (2.3)	3 (2.0)	3 (4.3)	3 (2.1)	0.749

Data is presented as mean ± SD or number (percentage)

NLR neutrophil to lymphocyte ratio, PLR platelet to lymphocyte ratio, TVR target vessel revascularization, TLR target lesion revascularization

*: p<0.05 compared with Low PLR, Low NLR

+: p<0.05 compared with Low PLR, High NLR

#: p<0.05 compared with High PLR, Low NLR

**Table 5:** Association between baseline clinical and angiographic characteristics and outcomes of the study population and neutrophil-to-lymphocyte ratio (NLR) or platelet-to-lymphocyte ratio (PLR).

	NLR		PLR	
	r	p	r	p
Age	0.081	0.021	0.106	0.003
Female gender	-0.040	0.262	0.077	0.029
Body mass index	-0.162	<0.001	-0.126	0.001
Current smoking	0.021	0.547	0.145	<0.001
Hypertension	0.009	0.800	0.004	0.920
Diabetes mellitus	0.029	0.408	0.025	0.479
Dyslipidemia	0.035	0.329	0.050	0.154
eGFR	-0.065	0.065	-0.048	0.171
hs-CRP	0.238	<0.001	0.174	<0.001
Ejection fraction	-0.163	<0.001	-0.028	0.438
Number of DES	0.014	0.699	0.008	0.829
Stent diameter	0.116	0.001	0.202	<0.001
Stent length	0.013	0.719	-0.021	0.546
ACC/AHA B2C lesion	0.031	0.382	-0.006	0.866
All-cause mortality	0.168	<0.001	0.090	0.011
Cardiac death	0.168	<0.001	0.105	0.003
Nonfatal MI	0.075	0.034	0.039	0.269
Stroke	0.002	0.964	-0.002	0.959
TLR	0.028	0.436	0.016	0.657
TVR	-0.016	0.653	-0.001	0.980

NLR neutrophil to lymphocyte ratio, PLR platelet to lymphocyte ratio, eGFR estimated glomerular filtration rate according to the Modification of Diet in Renal Disease (MDRD) equation, hs-CRP high sensitivity C-reactive protein, DES drug eluting stent, MI myocardial infarction, TVR target vessel revascularization, TLR target lesion revascularization

**Fig 2:**
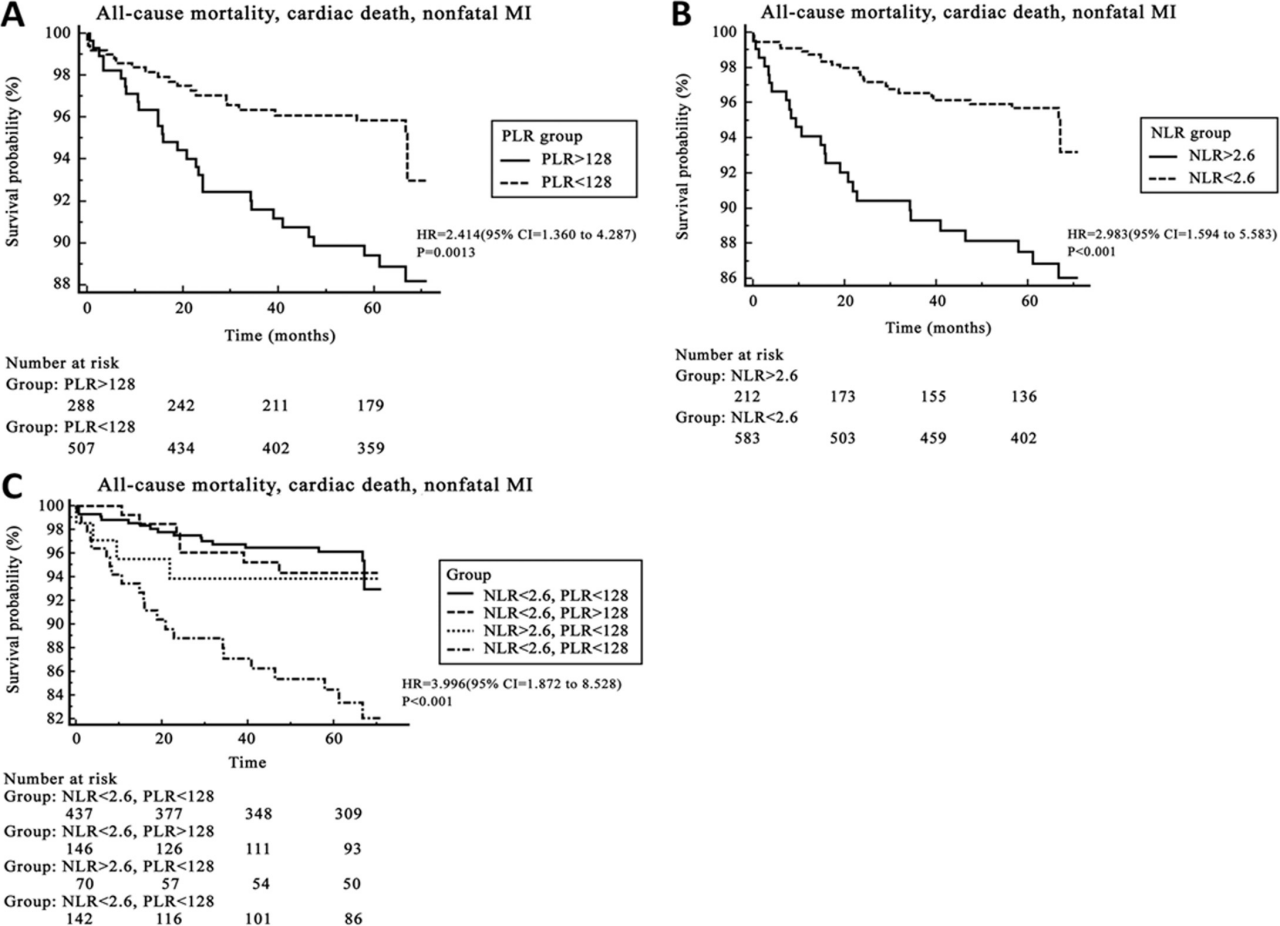
The Kaplan–Meier analysis shows the cumulative event-free composite rate of death and nonfatal myocardial infarction (MI) according to the optimal cut-off value of a PLR of 128 (A) and a NLR of 2.6 (B). Patients with a high PLR and NLR(C) showed the lowest long-term survival and clinical outcomes compared to the other groups. NLR; neutrophil-to-lymphocyte ratio, PLR; platelet-to-lymphocyte ratio.

Kaplan–Meier analysis revealed poor long-term survival and clinical outcomes in patients with a high PLR (PLR >128) (Fig 2A, hazard ratio HR 2.414, 95% CI 1.360 to 4.287, p = 0.0013) and a high NLR (NLR >2.6) (Fig 2B, HR 2.983, 95% CI 1.594 to 5.583, p <0.001). When patients with a combined high PLR and high NLR were examined, an even higher HR was observed (Fig 2C, HR 3.996, 95% CI 1.872 to 8.528, p <0.001). On Cox univariate analysis showed that hs-CRP (p = 0.002), NLR (p< 0.001), PLR (p = 0.07), eGFR (p < 0.001), LV ejection fraction (p < 0.001), hypertension (p = 0.003) and DM (p = 0.030) were significant predictors. On Cox multivariate analysis, a high NLR > 2.6 (HR, 2.352, 95% CI, 1.286 to 4.339, p = 0.006) and a high PLR >128 (HR 2.372, 95% CI 1.305 to 3.191, p = 0.005) were found to be independent predictors of long-term adverse events, and the combination of a high PLR and NLR was the strongest predictor of adverse events (HR 2.686, 95% CI 1.452 to 4.970, p = 0.002, Table 6). Additional significant independent predictors were increased hs-CRP, presence of hypertension and reduced LV ejection fraction (Table 6). When we performed sensitivity analysis separately for stable angina vs. ACS as the multivariate analysis, the results for ACS were consistent with those for the total population, however, for stable angina, none of the parameters are valuable for predicting adverse events (S1 and S2 Tables).

**Table 6:** Predictors of composite endpoint (all-cause mortality, cardiac death and non-fatal MI) by multivariate Cox regression analysis.

	HR	95% CI	p-value
Model 1			
Hs-CRP	1.120	1.062 to 1.182	<0.001
Age	1.019	0.977 to 1.056	0.421
Estimated GFR	0.976	0.951 to 1.001	0.057
Hypertension	2.488	1.061 to 5.835	0.036
Diabetes mellitus	1.240	0.563 to 2.731	0.594
Ejection fraction	0.019	0.001 to 0.380	0.010
Model 2			
PLR>128	2.372	1.305 to 3.191	0.021
Age	1.013	0.981 to 1.045	0.443
Estimated GFR	0.987	0.968 to 1.007	0.198
Hypertension	2.269	1.180 to 4.362	0.014
Diabetes mellitus	1.473	0.781 to 2.779	0.232
Ejection fraction	0.002	0.000 to 0.028	<0.001
Model 3			
NLR>2.6	2.352	1.286 to 4.339	0.006
Age	1.012	0.981 to 1.043	0.467
Estimated GFR	0.987	0.968 to 1.006	0.191
Hypertension	2.258	1.167 to 4.369	0.016
Diabetes mellitus	1.442	0.764 to 2.718	0.258
Ejection fraction	0.003	0.000 to 0.049	<0.001
Model 4			
NLR>2.6 and PLR>128	2.686	1.452 to 4.970	0.002
Age	1.013	0.982 to 1.045	0.422
Estimated GFR	0.989	0.969 to 1.008	0.252
Hypertension	2.165	1.124 to 4.168	0.021
Diabetes mellitus	1.442	0.767 to 2.713	0.256
Ejection fraction	0.002	0.000 to 0.046	<0.001

Hs-CRP high sensitivity C-reactive protein, NLR neutrophil to lymphocyte ratio, PLR platelet to lymphocyte ratio, MI myocardial infarction, HR hazard ratio, CI confidence interval, GFR glomerular filtration rate

## Discussion

In the present study, we showed that a high NLR and a high PLR on admission are independent predictors of long-term adverse events after successful PCI with DES in patients with ACS (unstable angina and NSTEMI). Additionally, our results showed that the combination of a high PLR and a high NLR is even more strongly predictive of future adverse events. To the best of our knowledge, this is the first study demonstrating the combined usefulness of the PLR and NLR for predicting the long-term adverse outcomes in patients who have undergone PCI with DES.

There has been rapidly growing interest in the association between the NLR and the risk of cardiovascular events in patients undergoing angiography or cardiac revascularization [5–9]. Recently, a meta-analysis of 10 cohort studies showed significant evidence to support the association between a high NLR and an increased risk of all-cause mortality and cardiovascular events [17]. A possible pathophysiological explanation for this relationship is the role of neutrophils in the mediation of the inflammatory response to acute myocardial injury resulting in further tissue damage [7]. Numerous biochemical mechanisms including the release of reactive oxygen species, myeloperoxidase, and proteolytic enzymes facilitate plaque disruption [18,19]. Lymphocytes are involved in the regulatory pathway of the immune system [20] and inflammation increases lymphocyte apoptosis [21]. Therefore, a composite marker of inflammation reflecting high neutrophils and low lymphocytes may provide additive information in the assessment of cardiovascular risk [22]. Most recent trials have targeted the role of the NLR in the long-term outcomes in patients with STEMI undergoing primary PCI [8,9], however, in the present study, we aimed to establish the independent role of the inflammatory markers in cardiovascular outcomes even after elective PCI. So we examined the role of the NLR and PLR in predicting the long-term adverse events in elective patients receiving PCI with DES, and excluded patients with STEMI receiving primary PCI. In our study, we confirmed the predictive value of NLR and PLR in the long term cardiovascular outcomes in patients with unstable angina and NSTEMI, who represent a lesser extent inflammatory response compared to those with STEMI. Taking into consideration the current increase in DES use, our study offers a unique perspective on the combined usefulness of the NLR and PLR in predicting the long-term adverse outcomes of PCI with DES for angina and non-ST-segment elevation MI patients.

Recently, the PLR, another marker of inflammation, has been evaluated as a prognostic marker in many cardiovascular diseases such as hypertension, CAD, and occlusive peripheral arterial disease [11–13]. Although the association between the NLR and cardiovascular disease has been demonstrated in numerous studies, the association between the PLR and cardiovascular disease remains unclear, apart from findings from a few clinical studies [14,15, 23,24]. Azab et al. [14] showed that a higher PLR was a significant independent marker of long-term mortality in patients with NSTEMI, while Kurtul et al. [15] showed an association between high PLR and no reflow phenomena in patients undergoing primary PCI [15]. Similar to the NLR, an elevated PLR was also associated with a significant increase in all-cause mortality risk and cardiovascular events after PCI [23, 24]. One possible explanation for the relationship between the PLR and cardiovascular events is an increased inflammatory response. Platelets can increase in number in response to various stimuli such as systemic infection, inflammatory conditions, bleeding, and tumors as acute phase reactants, which can result in the overproduction of pro-inflammatory cytokines that stimulate megakaryocytic proliferation and produce a relative thrombocytosis [25,26]. Higher platelet counts may reflect underlying inflammation and lower lymphocyte counts may represent an uncontrolled inflammatory pathway. Thus, a higher PLR may be a useful inflammatory marker [27]. Another possible mechanism could be that the high platelets represent a prothrombotic state, which is speculated to be a precursor of thrombosis [28]. Higher platelet counts may represent a higher propensity to form platelet-rich thrombi in atherosclerotic plaques, which may lead to worse outcomes. Although the severity of renal insufficiency is known to increase cardiovascular morbidity and mortality [29], the renal insufficiency was not an independent predictor in our study after adjustment of inflammatory markers. The mechanisms by which renal insufficiency adversely affect the poor results of reperfusion therapy of ACS include the stimulation of oxidative stress and inflammation by uremic toxins which may contribute to endothelial dysfunction and atherosclerosis progression [30]. However, as we excluded patients with a history of chronic renal disease, the impact of renal insufficiency on the long-term outcome has not been clearly elucidated in the present study. Another factor such as the admission or discharge medications which are known to improve survival after MI such as anti-platelets, beta-blockers, renin-angiotensin-aldosterone system blockers, and statins also did not influence the long-term outcomes in the present study.

Interestingly, from our results, hs-CRP levels were found to be significantly associated with both the NLR and PLR, and were the highest in patients with a high PLR and a high NLR. Moreover, hs-CRP level was also a significant independent predictor for the long-term adverse events in our study. Traditionally, hs-CRP has been well established as an inflammatory marker. However, in our experience, measuring the NLR and PLR is more cost-effective and frequently used than measuring hs-CRP. Considering the thrombosis and inflammation in patients with ACS might be different from those of stable angina [31], we performed sensitivity analysis separately for stable angina vs. ACS as the multivariate analysis, and a high NLR and a high PLR on admission were noted as independent predictors of long-term adverse events after successful PCI with DES only in patients with ACS, and not in those with stable angina.

This study had several limitations. Firstly, the NLR and PLR were based on a single measurement. It would be interesting to see if the NLR and PLR changed over time or if on subsequent tests they remain a predictor of CAD severity. Secondly, despite not crucial to the aim of this study, use of first generation stents only may have been a limitation [32]. Moreover, we did not evaluate the status of HIV infection in our patients, because of the rare prevalence of HIV in Korea. However, considering HIV related inflammation has shown to have a significant influence on the results [33], a lack of these data may be a limitation. More than 10% (n = 112) of the initially enrolled patients were lost to follow-up, and this would be a limitation as well. Finally, we excluded patients with renal or hepatic impairment, previous PCI, coronary artery bypass graft or stroke and therefore, this represents a very selective population.

### Conclusions

In conclusion, a high pre-intervention PLR and NLR, especially in combination, are independent predictors of long-term adverse clinical outcomes such as all-cause mortality, cardiac deaths, and myocardial infarction in patients with unstable angina and NSTEMI who underwent successful PCI with DES.

## Supporting Information

S1 Table Predictors of composite endpoint (all-cause mortality, cardiac death and non-fatal MI) in patients with unstable angina and non-ST elevated MI by multivariate Cox regression analysis. (DOCX)

S2 Table Predictors of composite endpoint (all-cause mortality, cardiac death and non-fatal MI) in patients with stable angina by multivariate Cox regression analysis. (DOCX)
