# Supplementary material for: Combined Usefulness of the Platelet-to-Lymphocyte Ratio and the Neutrophil-to-Lymphocyte Ratio in Predicting the Long-Term Adverse Events in Patients Who Have Undergone Percutaneous Coronary Intervention with a Drug-Eluting Stent
Source: PLoS One. 2015 Jul 24;10(7):e0133934. doi: 10.1371/journal.pone.0133934 (PMC4514869; doi:10.1371/journal.pone.0133934)
Supplement: S1 Table — (DOCX) [file pone.0133934.s001.docx]

**S1 Table.** Predictors of composite endpoint (all-cause mortality, cardiac death and non-fatal MI) in patients with unstable angina and non-ST elevated MI by multivariate Cox regression analysis

|  | HR | 95% CI | p-value |
| --- | --- | --- | --- |
| Model 1 | | | |
| Hs-CRP | 1.114 | 1.054 to 1.178 | <0.001 |
| Age | 1.015 | 0.974 to 1.056 | 0.486 |
| Estimated GFR | 0.978 | 0.951 to 1.005 | 0.115 |
| Hypertension | 2.115 | 0.875 to 5.109 | 0.096 |
| Diabetes mellitus | 1.303 | 0.553 to 3.074 | 0.545 |
| Ejection fraction | 0.008 | 0.000 to 0.031 | <0.001 |
| Model 2 | | | |
| PLR>128 | 2.358 | 1.251 to 4.442 | 0.008 |
| Age | 1.010 | 0.977 to 1.044 | 0.562 |
| Estimated GFR | 0.986 | 0.966 to 1.007 | 0.203 |
| Hypertension | 2.001 | 1.017 to 3.937 | 0.045 |
| Diabetes mellitus | 1.394 | 0.705 to 2.758 | 0.340 |
| Ejection fraction | 0.002 | 0.000 to 0.031 | <0.001 |
| Model 3 | | | |
| NLR>2.6 | 2.391 | 1.255 to 4.554 | 0.008 |
| Age | 1.013 | 0.983 to 1.043 | 0.549 |
| Estimated GFR | 0.986 | 0.966 to 1.007 | 0.191 |
| Hypertension | 1.968 | 0.994 to 3.897 | 0.052 |
| Diabetes mellitus | 1.363 | 0.689 to 2.698 | 0.374 |
| Ejection fraction | 0.003 | 0.000 to 0.053 | <0.001 |
| Model 4 | | | |
| NLR>2.6 and PLR>128 | 2.741 | 1.429 to 5.257 | 0.002 |
| Age | 1.012 | 0.979 to 1.046 | 0.490 |
| Estimated GFR | 0.988 | 0.967 to 1.009 | 0.251 |
| Hypertension | 1.878 | 0.953 to 3.701 | 0.069 |
| Diabetes mellitus | 1.357 | 0.687 to 2.682 | 0.379 |
| Ejection fraction | 0.003 | 0.000 to 0.050 | <0.001 |

Hs-CRP high sensitivity C-reactive protein, NLR neutrophil to lymphocyte ratio, PLR platelet to lymphocyte ratio, MI myocardial infarction, HR hazard ratio, CI confidence interval, GFR glomerular filtration rate
