# Supplementary material for: Combined Usefulness of the Platelet-to-Lymphocyte Ratio and the Neutrophil-to-Lymphocyte Ratio in Predicting the Long-Term Adverse Events in Patients Who Have Undergone Percutaneous Coronary Intervention with a Drug-Eluting Stent
Source: PLoS One. 2015 Jul 24;10(7):e0133934. doi: 10.1371/journal.pone.0133934 (PMC4514869; doi:10.1371/journal.pone.0133934)
Supplement: S2 Table — (DOCX) [file pone.0133934.s002.docx]

**S2 Table.** Predictors of composite endpoint (all-cause mortality, cardiac death and non-fatal MI) in patients with stable angina by multivariate Cox regression analysis

|  | HR | 95% CI | p-value |
| --- | --- | --- | --- |
| Model 1 | | | |
| Hs-CRP | 1.991 | 0.788 to 5.030 | 0.145 |
| Age | 1.067 | 0.889 to 1.281 | 0.485 |
| Estimated GFR | 0.972 | 0.948 to 1.056 | 0.397 |
| Hypertension | 4.383 | 0.514 to 37.3 | 0.176 |
| Diabetes mellitus | 5.645 | 0.488 to 65.3 | 0.166 |
| Ejection fraction | 0.000 | 0.000 to 74.61 | 0.457 |
| Model 1 | | | |
| PLR>128 | 2.418 | 0.362 to 16.15 | 0.362 |
| Age | 1.031 | 0.926 to 1.148 | 0.583 |
| Estimated GFR | 1.001 | 0.948 to 1.056 | 0.981 |
| Hypertension | 4.087 | 0.427 to 39.14 | 0.222 |
| Diabetes mellitus | 5.077 | 0.817 to 31.55 | 0.081 |
| Ejection fraction | 0.000 | 0.000 to 74.61 | 0.362 |
| Model 2 | | | |
| NLR>2.6 | 3.230 | 0.381 to 27.35 | 0.282 |
| Age | 1.012 | 0.913 to 1.121 | 0.937 |
| Estimated GFR | 0.998 | 0.947 to 1.051 | 0.937 |
| Hypertension | 5.532 | 0.462 to 66.22 | 0.177 |
| Diabetes mellitus | 5.673 | 0.846 to 38.05 | 0.537 |
| Ejection fraction | 0.001 | 0.381 to 27.35 | 0.286 |
| Model 3 | | | |
| NLR>2.6 and PLR>128 | 3.798 | 0.423 to 34.09 | 0.233 |
| Age | 1.011 | 0.914 to 1.118 | 0.835 |
| Estimated GFR | 1.001 | 0.949 to 1.055 | 0.983 |
| Hypertension | 5.947 | 0.481 to 73.49 | 0.165 |
| Diabetes mellitus | 5.744 | 0.868 to 38.03 | 0.070 |
| Ejection fraction | 0.001 | 0.000 to 170.9 | 0.270 |

Hs-CRP high sensitivity C-reactive protein, NLR neutrophil to lymphocyte ratio, PLR platelet to lymphocyte ratio, MI myocardial infarction, HR hazard ratio, CI confidence interval, GFR glomerular filtration rate
